# Efficacy of Watercress (*Nasturtium officinale* R.Br.) Consumption on Blood Pressure, Oxidative Stress Biomarkers, and Estimated Cardiovascular Risk in Thai Middle-Aged Adults: A Randomized Placebo-Controlled Pilot Study

**DOI:** 10.3390/antiox15060766

**Published:** 2026-06-19

**Authors:** Praporn Kijkuokool, Kittipan Rerkasem, Puriwat Fakfum, Wason Parklak, Hataichanok Chuljerm, Wiritphon Khiaolaongam, Chikondi Maluwa, Kanokwan Kulprachakarn

**Affiliations:** 1 School of Health Sciences Research, Research Institute for Health Sciences, Chiang Mai University, Chiang Mai 50200, Thailand; praporn_k@cmu.ac.th (P.K.); kittipan.r@cmu.ac.th (K.R.); puriwat_f@cmu.ac.th (P.F.); wason.p@cmu.ac.th (W.P.); hataichanok.ch@cmu.ac.th (H.C.); wiritphon_k@cmu.ac.th (W.K.); chikondi_maluwa@cmu.ac.th (C.M.); 2 Department of Surgery, Faculty of Medicine, Chiang Mai University, Chiang Mai 50200, Thailand

**Keywords:** watercress, *Nasturtium officinale* R.Br., cardiovascular disease, Framingham risk equation, blood pressure, oxidized LDL, antioxidant capacity

## Abstract

Watercress (*Nasturtium officinale* R.Br.) is a cruciferous vegetable rich in bioactive compounds that may improve cardiovascular disease (CVD) risk factors. However, clinical evidence regarding its direct impact on CVD risk remains limited. This study evaluated the efficacy of watercress consumption on cardiovascular parameters, oxidative stress biomarkers, and estimated CVD risk in middle-aged Thai adults with low-to-moderate risk. Twenty-five participants completed the randomized, placebo-controlled pilot study. The watercress group (*n* = 12) consumed 16 dried watercress capsules daily for four weeks, while the placebo group (*n* = 13) received a placebo. Physical examinations, arterial stiffness, lipid profiles, and biochemical biomarkers were analyzed at baseline and the end of treatment. The 10-year CVD risk was estimated using the Framingham equation. Following the intervention, the watercress group showed significant reductions in both systolic and diastolic blood pressure compared to the placebo group. Within the watercress group, significant improvements from baseline to post-intervention were observed in oxidized LDL, antioxidant capacity (ABTS), and estimated 10-year CVD risk score. However, these three parameters did not reach statistical significance when compared to the placebo group. In conclusion, daily watercress consumption significantly lowers blood pressure and demonstrates a potential dietary option for supporting cardiovascular health. Nevertheless, larger and longer-term clinical trials remain necessary.

## 1. Introduction

Cardiovascular diseases (CVDs) are currently the major cause of global mortality. The number of global deaths due to CVDs increased from 13.1 million in 1990 to 19.2 million in 2023. Moreover, CVDs were responsible for 437 million disability-adjusted life years (DALYs) in that year [1]. This burden is equally reflected in Thailand, where CVD mortality rose from 82,220 in 2000 to 114,807 in 2019, accounting for 23% of all mortality in that year [2]. The overall CVD mortality eventually reached 136,761 in 2021 [3]. The risk factors for CVDs include metabolic syndromes, such as hypertension, hyperlipidemia, diabetes, and obesity, as well as unhealthy lifestyles characterized by unhealthy diet, tobacco use, alcohol consumption, and physical inactivity [2]. The effective management of CVD risk factors is essential to prevent and postpone the onset of CVDs.

Several previous reviews indicated that pharmacotherapy, including antihypertensive and lipid-modifying agents, effectively reduced CVD events or mortality rates. These pharmacotherapies are advantageous for individuals with intermediate or high risk of CVDs [4]. Beyond their primary effects on blood pressure (BP) or lipid profiles, these medications mitigate CVD risk and CVD events through various pleiotropic mechanisms [5]. However, the requirement for consistent clinical supervision and the potential for adverse effects highlight the importance of exploring non-pharmacological alternatives.

Lifestyle modification is widely recognized as the first-line intervention for CVD management. It is low-cost and can be conducted across various settings. One of the lifestyle management strategies involves consuming a diet rich in vegetables, fruit, whole grains, fiber, and antioxidants, while limiting the intake of salt, sugar, saturated fat, and trans fat [4,6]. Furthermore, the National Nutrition Guidelines advocate for the consumption of powerhouse fruits and vegetables (PFV). PFV is a food that contains high nutrient density, including 17 essential nutrients, and is closely linked to the reduction of chronic disease risk. Notably, watercress ranks as the highest-scoring PFV among the 47 studied PFV lists [7].

Watercress (*Nasturtium officinale* R.Br.) is a semi-aquatic perennial leafy vegetable belonging to the Brassicaceae family, natively distributed across Europe, Asia, India, and Africa [8]. Watercress has been widely found in the southern region of Thailand, specifically the Betong district in Yala province, where it is locally referred to as “Betong watercress” [9]. It is characterized by a rich profile of bioactive phytochemicals, particularly glucosinolates, isothiocyanate derivatives, dietary nitrates, phenolic acids, flavonoids, proanthocyanidins, monoterpenoids, sesquiterpenoids, diterpenoids, and tetraterpenoids [10,11,12]. The presence of these diverse secondary metabolites contributes to numerous therapeutic benefits. Watercress has historically been used in traditional medicine to enhance overall health. It has been used to ameliorate asthma, cough, and bronchial issues [11]. Extensive clinical research and pharmacological reviews have demonstrated that watercress acts as a potent antioxidant and anti-inflammatory agent, protecting cells from oxidative damage and reducing systemic inflammation. It contributes to metabolic syndrome by improving lipid profiles, lowering blood glucose, and enhancing insulin sensitivity. Additionally, watercress demonstrates anticancer, nephroprotective, and hepatoprotective characteristics, as well as impacts on chronic respiratory conditions [11,13,14,15,16,17].

Despite the documented therapeutic potential of watercress in managing individual metabolic disorders, studies investigating its direct impact on CVD risk in humans remain unclear. Therefore, this pilot study aimed to evaluate the protective efficacy of watercress against CVD-related risk factors and to determine its effectiveness in reducing CVD risk.

## 2. Materials and Methods

### 2.1. Watercress and Placebo Treatments

A single batch of fresh watercress was purchased from Zhi Wu Watercress Farm in Yala province, Thailand, in December 2024. Watercress was then roasted at 50 °C, ground, and packed in a size 0 hard gelatin capsule (500 mg).

The dosage of watercress used in the previous study was varied according to the format of use. In the form of fresh vegetables, the dosage of watercress could be 85 g/d [18,19,20] or 180 g/d [21]. Regarding the extract form, the dosage could be 500 mg/d [22] or 750 mg/kg/d with 5.0 mg/mL of phenylethyl glucosinolate [23,24]. In the current study, glucosinolate is the major phytochemical of interest as well. Our previous study demonstrated that dried watercress (using the roasting method) provided a total glucosinolate content of 24.34 mg/g dry weight, as quantified via a colorimetric spectrophotometric assay (BMG LABTECH, Ortenberg, Germany) at an absorbance of 425 nm using a sinigrin standard calibration curve (0.05–2 mg/mL) (Sigma-Aldrich, Darmstadt, Germany) [25]. Therefore, the final dosage of dried watercress in this study was 8 g/d (or 16 capsules/d), which contained a total glucosinolate of 195 mg/d.

Regarding the placebo, cornstarch was packed in a 500 mg capsule [26]. The drying process of watercress, along with the packaging, was conducted by Tungsuwan Organic Farm Co., Ltd., Chiang Mai, Thailand.

### 2.2. Human Ethical Approval

This pilot study was approved by the Human Experimentation Committee (HEC) of the Research Institute for Health Sciences, Chiang Mai University (Project No. 22/67) on 10 October 2024. The protocol was registered at www.thaiclinicaltrials.org (TCTR20251119004) on 19 November 2025.

### 2.3. Participants

Participants were included based on the specific inclusion and exclusion criteria. Eligible individuals were required to be middle-aged Thai adults (aged 40–59 years) with low or moderate risk of cardiovascular diseases (less than 15% of 10-year CVD risk, according to the Framingham risk equation [27]) and have at least one of the following conditions [28]: systolic blood pressure (SBP) of 130–160 mmHg, total cholesterol (TC) of 200–240 mg/dL, fasting blood glucose (FBG) of 100–125 mg/dL, body mass index (BMI) of 25–40 kg/m^2^, or waist circumference greater than 90 cm for males or greater than 80 cm for females. Additionally, individuals with chronic diseases or psychiatric conditions who were on medication, as well as pregnant or lactating women, were excluded. All participants were fully informed about the study procedures and then completed the informed consent form prior to enrollment.

### 2.4. Sample Size

The sample size for comparing the means of two independent groups was calculated using Equation (1). Based on the previous study of Shokraei et al. [29], the difference in the mean of high-density lipoprotein cholesterol (HDL-C) of the watercress group and the control group was 2.7 mg/dL, with the SDs of 2.0 and 2.2. The sample size was determined using a two-sided 95% confidence level (z_α_ = 1.96) and 80% statistical power (z_β_ = 0.84). The calculated sample size was 10 participants per group. After combining with a 25% dropout rate, the final sample size was 13 participants in each group, or a total of 26 participants.

(1) $$n=\frac{{\left(z_{\alpha }+z_{ꞵ}\right)}^{2}({\sigma_{1}}^{2}+{\sigma_{0}}^{2})}{{\left({µ}_{1}-{µ}_{0}\right)}^{2}}$$

### 2.5. Study Design

This randomized, single-blinded, placebo-controlled pilot study was conducted between January and March 2025 to evaluate the effects of the 4-week consumption of dried watercress on CVD risk factors.

One week before the beginning of the study, a total of 27 volunteers were fully informed about the study procedures and performed the screening test, including weight, height, and waist circumference measurements and blood pressure monitoring. Their fasting blood was collected to examine the levels of FBG, TC, and HDL-C. The 10-year CVD risk score was calculated using the Framingham equation. Following the screening process, one participant was excluded for not meeting the eligibility criteria, leaving 26 participants for randomization. The remaining participants signed the written informed consent before participating in the study.

Twenty-six participants were randomly divided into two groups using computer-generated block randomization. To ensure allocation concealment, an independent researcher uninvolved in participant recruitment generated the randomization sequence. Furthermore, the intervention capsules were packaged in sequentially numbered, opaque, sealed containers, which were delivered to the participants only post-enrollment. The experimental group (*n* = 13), called the watercress group (W group), received dried watercress packed in 500 mg capsules. They were assigned to consume 4 capsules after every meal and before going to bed (4 times per day). According to this amount of watercress, they received 195 mg of glucosinolate per day. The placebo group (P group) was assigned to consume 4 cornstarch capsules after every meal and before going to bed (4 times per day). The color of the capsule for both watercress and cornstarch was dark green; therefore, the participants were blinded and were unaware of their treatment. The participants were assigned to consume the capsules consecutively for 4 weeks [22]. All participants were asked to maintain their usual lifestyle patterns (physical exercise, diet, and sleeping habits) during the intervention period. The investigator called the participants twice a week to monitor compliance and any adverse effects after receiving the treatment. If any adverse event was reported, the researchers would assess the causality of product consumption using Naranjo’s algorithm [30,31] and would promptly consult a physician for further medical evaluation. Due to the nature of the intervention, not all interventionists could be blinded. However, outcome assessors, including clinical staff responsible for physiological examination and the investigator who contacted participants, remained blinded to group allocation throughout the study.

**Figure 1:**
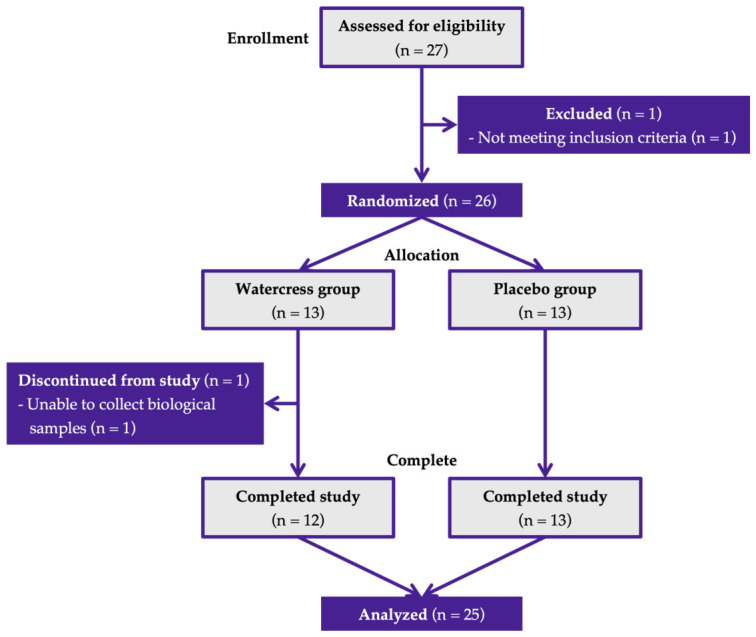
Participant flow diagram for the randomized, single-blinded, placebo-controlled pilot study.

At baseline (day 1) and at the end of treatment (EOT, day 29), the participants were scheduled for a physical examination and blood collection. Venous blood samples were collected after a 12-h fast. The physical examinations included weight, height, waist and hip circumferences, and vital signs. Cardio-ankle vascular index (CAVI) and ankle-brachial index (ABI) were also performed to measure arterial stiffness. During the final visit, one participant from the experimental group was withdrawn due to the inability to collect biological samples (blood). Consequently, twenty-five participants completed the study, consisting of 12 in the W group and 13 in the P group (Figure 1).

### 2.6. Biochemical Analysis

The collected blood samples were used to evaluate FBG and lipid profiles. All blood samples were processed and analyzed at the Bangkok R.I.A. LAB Co., Ltd. (Chiang Mai, Thailand). Serum was separated by centrifugation and stored at −80 °C for the subsequent analysis of oxidative stress and antioxidant biomarkers. Oxidized low-density lipoprotein (oxLDL) levels were determined in serum using an ELISA kit (FineTest^®^, Wuhan, China). The antioxidant capacity was evaluated in serum using the 2,2′-azino-bis-(3-ethylbenzothiazoline-6-sulfonic acid) (ABTS) radical cation decolorization assay adapted from previous studies (Sigma-Aldrich, Darmstadt, Germany) [32,33].

### 2.7. Statistical Analysis

All statistical analyses were performed by IBM SPSS Statistics for macOS Version 29. The results were expressed as the percentage, mean, standard deviation (SD), median, and interquartile range (IQR). The normality of the data was evaluated using the Shapiro–Wilk test. Non-parametric analysis of variance was used to analyze the data. The comparison between baseline and EOT data within each group was analyzed by the Wilcoxon signed-rank test. For between-group comparisons, the Mann–Whitney U test was independently applied at two specific time points (baseline and EOT) to compare the W and P groups. To quantify the precision of the observed effects, 95% confidence intervals (CIs) for the differences were calculated using the Hodges-Lehmann estimation, and effect sizes (*r*) were determined for all primary endpoints. A *p*-value of less than 0.05 is considered significant.

## 3. Results

### 3.1. Baseline Characteristics of Participants

The baseline characteristics of the participants are summarized in Table 1. The W group (*n* = 12) consisted of an equal distribution of males (*n* = 6, 50%) and females (*n* = 6, 50%), with an average age of 48.42 ± 5.16 years. While there were 6 males (46.2%) and 7 females (53.8%) in the P group (*n* = 13), with a mean age of 47.31 ± 5.71 years. Regarding educational status, the majority of the participants (92%) had achieved a high school diploma or a higher degree. In terms of occupation, 48% were private company employees, while 44% were employed in government agencies. Additionally, four participants (16%) reported that they were current smokers, with an equal distribution between the W group (*n* = 2) and the P group (*n* = 2). There were no significant differences in baseline characteristics between the two groups (*p* > 0.05).

### 3.2. Cardiovascular Health Indicators

**Figure 2:**
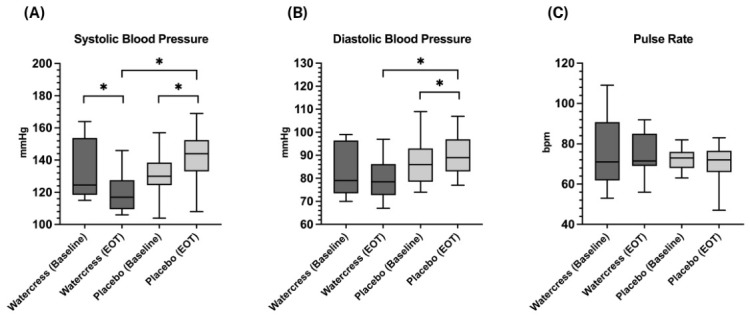
Baseline and EOT blood pressure of participants in the watercress and placebo groups. (**A**) Systolic blood pressure; (**B**) Diastolic blood pressure; (**C**) Pulse rate. Data are median (IQR). The comparison between baseline data and EOT data in each group was analyzed by the Wilcoxon signed-rank test. The comparison across two groups was analyzed by the Mann–Whitney U test. * Significantly different (*p*-value < 0.05). EOT, end of treatment; IQR, interquartile range.

**Figure 3:**
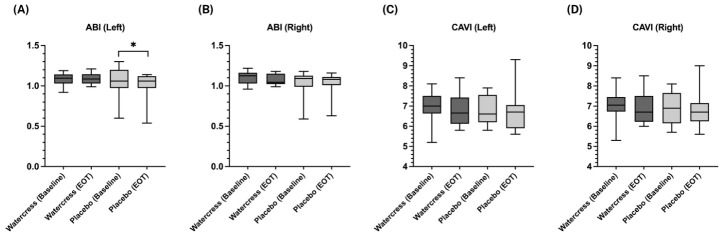
Baseline and EOT ABI and CAVI values of participants in the watercress and placebo groups. (**A**) ABI (left side); (**B**) ABI (right side); (**C**) CAVI (left side); (**D**) CAVI (right side). Data are median (IQR). The comparison between baseline data and EOT data in each group was analyzed by the Wilcoxon signed-rank test. The comparison across two groups was analyzed by the Mann–Whitney U test. * Significantly different (*p*-value < 0.05). ABI, ankle-brachial index; CAVI, cardio-ankle vascular index; EOT, end of treatment; IQR, interquartile range.

**Figure 4:**
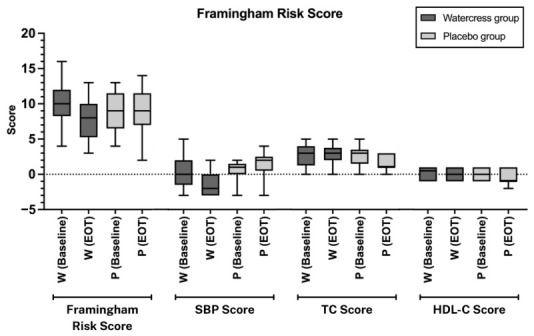
Baseline and EOT Framingham risk scores, including total score, SBP score, TC score, and HDL-C score, of participants in the watercress and placebo groups. Data are median (IQR). W, watercress group; P, placebo group; EOT, end of treatment; SBP, systolic blood pressure; TC, total cholesterol; HDL-C, high-density lipoprotein cholesterol; IQR, interquartile range.

The effects of watercress consumption on cardiovascular health indicators such as blood pressure, arterial stiffness, and CVD-risk score are presented in Figure 2, Figure 3 and Figure 4. After four weeks of treatment, the post-intervention SBP in the W group [median (IQR): 117.0 (109.5–127.5) mmHg] was significantly lower than that in the P group [144.0 (133.0–152.5) mmHg] (95% CI: 11.00, 34.00, *r* = 0.60, *p* = 0.002). Similarly, the EOT diastolic blood pressure (DBP) was significantly lower in the W group [78.5 (72.8–86.3) mmHg] compared to the P group [89.0 (83.0–97.0) mmHg] (95% CI: 2.00, 18.00, *r* = 0.51, *p* = 0.010). The intra-group comparison indicated that four weeks of watercress consumption significantly decreased SBP from the baseline level (95% CI: −27.00, −6.00, *r* = 0.86, *p* = 0.001). Conversely, the P group exhibited significant increases in both SBP and DBP at EOT compared to baseline (95% CI: 0.00, 24.00, *r* = 0.72, *p* = 0.007 and 95% CI: −3.00, 10.00, *r* = 0.55, *p* = 0.046, respectively). However, no significant difference was observed in pulse rate for both intercomparison and intra-group comparison, as presented in Figure 2.

Regarding other cardiovascular parameters, no significant changes were observed in the CAVI or ABI between baseline and EOT in each group (Figure 3). Except for the left-sided ABI in the P group, which exhibited a significant change (95% CI: −0.10, −0.02, *r* = 0.62, *p* = 0.022). Furthermore, intergroup comparisons demonstrated no statistically significant differences in these indicators at either baseline or EOT.

**Figure 5:**
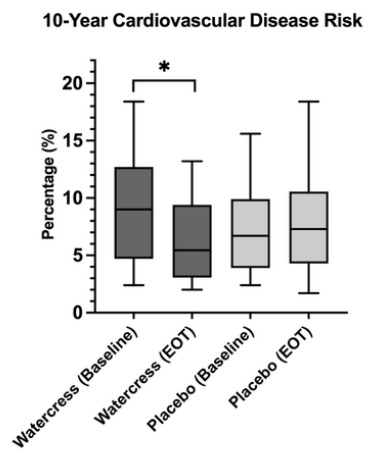
Baseline and EOT 10-year cardiovascular disease risk percentages of participants in the watercress and placebo groups. Data are median (IQR). The comparison between baseline data and EOT data in each group was analyzed by the Wilcoxon signed-rank test. The comparison across two groups was analyzed by the Mann–Whitney U test. * Significantly different (*p*-value < 0.05). EOT, end of treatment; IQR, interquartile range.

In terms of cardiovascular risk assessment, the Framingham risk equation was used to calculate the 10-year risk level. The important factors used in this calculation included age, gender, cholesterol level (TC and HDL-C), blood pressure, smoking status, and diabetes history. Figure 4 illustrates the distinct contributions of each metabolic parameter to CVD risk score, displaying the specific sub-scores (SBP, TC, and HDL-C) alongside the total risk scores at both baseline and EOT for each group. Concurrently, the percentage of 10-year CVD risk converted from these scores is displayed in Figure 5.

Following the 4-week intervention, the W group demonstrated a significant reduction in 10-year CVD risk, decreasing from 9.0% (4.7–12.7) at baseline to 5.5% (3.1–9.4) at EOT (95% CI: −5.20, −0.40, *r* = 0.80, *p* = 0.003). Conversely, no significant changes in the CVD risk percentage were observed within the P group. Between-group analysis showed no significant differences in Framingham risk percentages at baseline or at EOT, as shown in Figure 5.

### 3.3. Blood Biomarkers

The effects of the intervention on blood biomarkers are displayed in Table 2. After 4 weeks of consumption, the TC and triglyceride (TG) levels of the W group tended to decrease; however, no significant changes were observed. FBG and HDL-C levels in the W group exhibited no change either. However, low-density lipoprotein cholesterol (LDL-C) levels in this group showed a significant increase from a baseline of 166.0 (128.5–191.0) mg/dL to 175.5 (143.8–195.3) mg/dL at EOT (95% CI: 1.00, 32.00, *r* = 0.69, *p* = 0.014). In the P group, TC levels significantly decreased from 222.0 (202.5–246.0) mg/dL at baseline to 194.0 (176.0–225.5) mg/dL at EOT (95% CI: −42.00, −3.00, *r* = 0.80, *p* = 0.002), while no significant changes were found in FBG, TG, LDL-C, or HDL-C. Furthermore, intergroup comparisons revealed no statistically significant differences in any blood biomarkers at either baseline or EOT.

### 3.4. Oxidative Stress and Antioxidant Biomarkers

**Figure 6:**
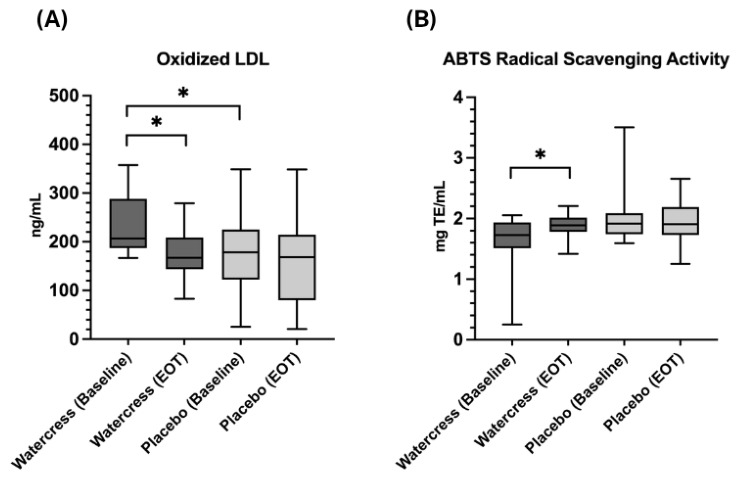
Baseline and EOT oxidative stress and antioxidant biomarkers of participants in the watercress and placebo groups. (**A**) Oxidized LDL; (**B**) ABTS radical scavenging activity. Data are median (IQR). The comparison between baseline data and EOT data in each group was analyzed by the Wilcoxon signed-rank test. The comparison across two groups was analyzed by the Mann–Whitney U test. * Significantly different (*p*-value < 0.05). LDL, low-density lipoprotein; ABTS, 2,2′-azino-bis-(3-ethylbenzothiazoline-6-sulfonic acid); TE, Trolox equivalent; EOT, end of treatment; IQR, interquartile range.

The findings regarding oxidative stress and antioxidant biomarkers are summarized in Figure 6. After the 4-week intervention, the W group showed a significant reduction in oxLDL levels compared to baseline level, decreasing from 206.95 (187.31–288.02) ng/mL to 167.19 (143.62–208.98) ng/mL (95% CI: −170.50, 3.81, *r* = 0.63, *p* = 0.027). Notably, this reduction represented a shift from elevated levels to within the clinically normal range (normal range: 10–170 ng/mL). Furthermore, the antioxidant capacity, measured by the ABTS assay, significantly increased in the W group from 1.73 (1.51–1.93) mg Trolox equivalent (TE)/mL at baseline to 1.89 (1.78–2.02) mg TE/mL at EOT (95% CI: −0.04, 0.64, *r* = 0.65, *p* = 0.027). In contrast, no significant changes in oxLDL or ABTS levels were observed in the P group. Despite these intra-group improvements, no statistically significant differences were found between the two groups at EOT.

## 4. Discussion

CVDs remain the leading cause of global and national mortality [34]. Therefore, accessible and effective prevention strategies are necessary. Dietary modification, specifically the increased intake of fiber and antioxidants, is one of the primary suggestions [4]. Among many nutrient-dense fruits and vegetables, watercress stands out, as it contains high concentrations of bioactive phytochemicals [7]. The present study evaluated the efficacy of a 4-week watercress intervention on biochemical profiles, antioxidant parameters, and cardiovascular risk factors among Thai individuals with low-to-moderate CVD risk. At baseline, cardiovascular parameters were compared across groups, with no significant differences observed in any baseline characteristic markers. This confirms that the study population was homogeneous and well-randomized.

Following the four-week intervention, watercress consumption demonstrated a favorable effect on blood pressure control. The SBP level in the W group was significantly reduced compared to both its baseline and that of the P group at post-intervention. Additionally, the DBP level exhibited a downward trend within the W group and was significantly lower than that of the P group at EOT. In contrast, no significant post-intervention changes were observed in pulse rate or arterial stiffness markers, CAVI and ABI.

The observed reduction in SBP and DBP is likely attributable to the high dietary nitrate content found in cruciferous vegetables. Watercress is classified as a moderate- to high-nitrate vegetable, with concentrations ranging from 500 to 2500 mg/kg [35,36,37]. For instance, Meamarbashi and Alipour [36] reported that raw watercress contained approximately 1095 mg/kg of nitrates, although these concentrations can fluctuate based on regional cultivation conditions [35].

Our findings align with previous clinical evidence suggesting the blood pressure-lowering efficacy of nitrate-rich supplements. For example, the randomized crossover trial by Hayes et al. [38] demonstrated that nitrate-rich beetroot juice, containing 14.0 mmol of nitrate, significantly reduced the SBP and DBP compared to a placebo at 2.5 h post-supplementation, accompanied by elevated plasma nitrate and nitrite concentrations. Similarly, Babateen et al. [39] investigated the effects of prolonged consumption of different doses of dietary nitrate. The finding indicated that supplementation with medium and low doses of nitrate for 13 weeks effectively lowered the SBP and enhanced endothelial function in the elderly.

These potential antihypertensive effects are hypothetically attributed to the well-established nitrate-nitrite-nitric oxide pathway. Upon ingestion, dietary nitrate (NO_3_^−^) is absorbed in the small intestine, enters the systemic circulation, and is subsequently concentrated by the salivary glands. In the oral cavity, bacteria (e.g., *Veillonella*, *Actinomyces*, *Rothia*, and *Staphylococcus epidermidis* species) converted salivary nitrate to nitrite (NO_2_^−^). Once swallowed, this nitrite undergoes protonation to form nitrous acid, which decomposes into nitric oxide (NO•) and other bioactive nitrogen oxides. The nitric oxide travels through the blood circulation and acts as a potent vasodilator. It diffuses into vascular smooth muscle cells, stimulating relaxation and reducing peripheral vascular resistance, thereby resulting in lower blood pressure [40,41,42,43].

Beyond the nitrate-nitrite-nitric oxide pathway, watercress may exert vascular benefits through the inhibition of angiotensin-converting enzyme (ACE). ACE is a key component of the renin–angiotensin–aldosterone system (RAAS), which mediates vasoconstriction, fluid balance, and elevated blood pressure [44]. An in vitro investigation by Yaricsha et al. [45] demonstrated that the ethanolic extract of watercress exhibited ACE inhibitory activity (IC_50_ value of 19.05 μg/mL), achieving a 34.34% inhibitory capacity relative to the standard antihypertensive agent, captopril. These mechanistic pathways likely provide an explanation for the potential blood pressure improvements observed in the current study.

In contrast to the significant reductions observed in blood pressure, the 4-week intervention showed no significant changes in CAVI or ABI scores between the groups. This lack of significant alteration may be attributed to the short intervention period, as structural improvements in arterial stiffness and peripheral arterial diameter typically require longer-term dietary modifications to become detectable. For instance, a study by Hamal et al. [46] demonstrated that three months of garlic extract consumption were required to significantly improve CAVI scores in patients with type 2 diabetes mellitus. Moreover, a clinical trial by Gylling et al. [47] reported no significant changes in CAVI even after six months of plant stanol ester consumption. This supports the idea that prolonged interventions are essential for vascular structural remodeling.

In addition to blood pressure regulation, the watercress intervention affected biomarkers associated with oxidative stress, observed only within the experimental group. Following the 4-week period, the W group exhibited a reduction in oxLDL levels alongside an increase in total antioxidant capacity. While these parameters did not reach statistical significance in the between-group analysis, the observed intra-group variations call for a mechanistic discussion regarding cardiovascular prevention. LDL oxidation is a critical early mechanism in the pathogenesis of atherosclerosis. The accumulation of oxLDL particles within the subendothelial space triggers endothelial activation, promoting monocyte recruitment and subsequent differentiation into macrophages. These macrophages engulf oxLDL to form foam cells, leading to the development of fatty streaks within the arterial wall [48,49].

Watercress is a well-known source of glucosinolates, which are enzymatically hydrolyzed into phenethyl isothiocyanate (PEITC) [50,51]. In cellular models, PEITC has been shown to exert protective effects against oxidative damage by upregulating nuclear factor erythroid 2-related factor 2 (Nrf2). For instance, Ko et al. [52] demonstrated that PEITC activated Nrf2-mediated gene expression in adipocyte models, leading to inhibition of adipocyte differentiation. Furthermore, Huang et al. [53] reported that PEITC and two other isothiocyanates, at concentrations of 0–10 μM in human umbilical vein endothelial cells (HUVECs), induced the expression of Nrf2-dependent heme oxygenase-1 (HO-1) and glutamate-cysteine ligase (GCL), while reversing oxLDL-induced reactive oxygen species (ROS) production.

Mechanistically, activated Nrf2 translocates to the nucleus and binds to antioxidant response elements (AREs), triggering the transcription of cytoprotective enzymes such as HO-1, NAD(P)H:quinone oxidoreductase (NQO1), superoxide dismutase (SOD), and glutathione peroxidase (GSH-Px). This endogenous defense system helps neutralize ROS before they can initiate lipid peroxidation [54]. In the present study, the intra-group increase in antioxidant capacity, as measured by the ABTS radical scavenging activity, aligns with this mechanistic framework, suggesting a potential priming of the systemic antioxidant defense system. Moreover, the antioxidant efficacy of watercress is likely augmented by the combined effects of other phytochemicals, such as flavonoids (quercetin, kaempferol, and rutin), phenolic acids, ascorbic acid (vitamin C), carotenoids (β-carotene, lutein, and zeaxanthin), and sulfur-containing compounds. These compounds function as direct free radical scavengers [13,14].

Alongside these cardiovascular observations, a notable alteration was detected in the lipid profiles regarding the statistically significant increase in LDL-C levels within the W group. This finding requires cautious interpretation, particularly since the between-group analysis showed no significant differences in lipid parameters at the end of treatment. A previous systematic review by Darand et al. [55] analyzed nine RCTs (including 548 participants) and demonstrated that Brassica vegetables had no significant impact on serum levels of TG, LDL-C, HDL-C, and FBG. Furthermore, a clinical study by Vacca et al. [56] involving twenty-six participants highlighted the role of individual metabolic variation. Following one month of kale consumption, individuals with a notable reduction in TC exhibited a significant decrease in LDL-C levels, whereas participants with stable TC levels demonstrated a significant increase in LDL-C.

This observed paradoxical elevation might be linked to acute shifts in hepatic lipid metabolism. High concentrations of specific dietary phytochemicals have been reported to temporarily modulate bile acid synthesis pathways [57], leading to a transient reduction in circulating LDL clearance. Consequently, further long-term, fully powered randomized controlled trials are required to clarify whether this observed increase represents a transient physiological adaptation or a clinically relevant metabolic trend.

Regarding the 10-year cardiovascular risk estimate, an intra-group score reduction was observed in the experimental group. The risk status shifted from the higher end of the “low-to-moderate” risk category to a “low” risk status. However, since the Framingham equation is intended for long-term risk estimation rather than short-term intervention assessment, these observations should be interpreted with caution. As the demographic variables (including age, sex, smoking status, and diabetes history) remained constant over the 4-week period, this score reduction was primarily driven by the significant reductions in SBP, the downward trend in TC, and the slight improvement in HDL-C levels. Consequently, these statistical shifts cannot imply that actual long-term cardiovascular risk was substantially reduced within the 4-week timeframe, particularly since no statistically significant differences in Framingham risk scores were detected between the W and P groups at the end of treatment.

While our short-term, small-scale pilot study cannot be directly compared to large-scale epidemiological data, the potential cardiovascular benefits of cruciferous vegetables are well-documented in broader contexts. For example, a 15-year cohort study of 1226 older Australian women by Blekkenhorst et al. [58] highlighted the potent cardioprotective effects of cruciferous vegetables. Despite an average baseline Framingham risk score of approximately 20%, the finding demonstrated that every 10-g daily increase in cruciferous vegetable intake could reduce the hazard of atherosclerotic vascular disease (ASVD) mortality by 13%. Although the large-scale cohort focused on long-term clinical endpoints, the phytochemical compounds discussed in their work provide possible mechanistic support for our findings. The organosulfur compounds (such as glucosinolates and isothiocyanate derivatives) may act as potent anti-inflammatory agents and antioxidants, thereby acutely improving endothelial function and blood pressure.

Despite the clinical observations reported in the current study, there are several limitations. First, the 4-week intervention duration may have been insufficient to fully capture the biological potential of watercress, as traditional dietary interventions often require 8–12 weeks to manifest alterations in lipid profiles. Second, this study was conducted as a small-scale pilot trial with a limited sample size, reducing the statistical power to detect significant differences between groups across multiple biochemical endpoints. Consequently, several interpretations were based mainly on intra-group changes, and formal adjustments for multiplicity were not applied. Furthermore, the use of the classic Framingham risk equation, which was developed in a Western population, may represent a predictive limitation when applied directly to a Thai cohort. Therefore, these preliminary statistical trends require further confirmation in adequately powered, long-term randomized controlled trials.

Next, the intervention utilized dried watercress powder in capsule form rather than fresh vegetables or concentrated extracts. While this approach ensured dosing, the comparative efficacy of different delivery formats remains to be elucidated. Moreover, this study is limited by the lack of a compositional profile for the watercress powder utilized, such as nitrate content, polyphenol characterization, or PEITC concentrations. Importantly, while the current study utilized a single batch of watercress to ensure consistency within this small cohort, batch-to-batch phytochemical variations cannot be confirmed due to the lack of high-performance liquid chromatography (HPLC) analysis. Therefore, the reproducibility of the observed clinical outcomes across different plant batches remains unverified until a standardized chemical fingerprint is established. Similarly, key mechanistic variables (including the concentrations of nitrate, nitrite, NO metabolites, ACE activity, inflammatory markers, and Nrf2 signaling variables) were not directly measured in the participants’ biological samples. Consequently, the involvement of these proposed biochemical pathways in our cohort remains speculative and is based on established literature rather than direct clinical outcomes. Additionally, while participants’ dietary patterns were monitored using 3-day dietary recalls, daily dietary intake and physical activity were not strictly controlled.

Finally, the intervention required participants to consume 16 capsules per day to achieve the target glucosinolate dosage. Although compliance remained high during this short-term pilot trial, the pill burden presents a practical obstacle to real-world feasibility and sustained long-term adherence. Future studies should focus on developing concentrated extracts to diminish capsule volume and improve clinical practicality.

Notably, despite the high capsule burden, no adverse effects or gastrointestinal (GI) symptoms were reported by any participants throughout the study period, suggesting excellent GI tolerability. Future powered, long-term randomized controlled trials are explicitly warranted to incorporate specific metabolite measurements, validate these proposed cardiovascular mechanisms, and determine the optimal chromatographic profiling and therapeutic dosage of watercress for clinical application.

## 5. Conclusions

In conclusion, this randomized, placebo-controlled pilot study demonstrated that a 4-week watercress intervention may potentially modulate specific cardiovascular risk factors in individuals with low-to-moderate CVD risk. Watercress consumption significantly reduced both SBP and DBP, hypothetically driven by the dietary nitrate-nitrite-nitric oxide pathway and potential ACE inhibitory activity. Furthermore, the intervention correlated with positive intra-group changes in systemic antioxidant capacity and a decline in oxLDL levels, which may be linked to PEITC-mediated Nrf2 activation. Although these short-term improvements did not lead to significant differences in long-term Framingham risk scores or macrovascular stiffness markers between the groups, our findings suggest that watercress represents a potential dietary option for supporting cardiovascular health. Nonetheless, further long-term, fully powered randomized controlled trials are required to validate these proposed mechanisms and establish definitive clinical efficacy.

## Figures and Tables

**Table 1:** Baseline characteristics of participants (*n* = 25).

Characteristic	Watercress Group (*n* = 12)	Placebo Group (*n* = 13)	Total (*n* = 25)
Age (years), Mean ± SD	48.42 ± 5.16	47.31 ± 5.71	47.84 ± 5.37
Gender, *n* (%)			
Male	6 (50.0)	6 (46.2)	12 (48.0)
Female	6 (50.0)	7 (53.8)	13 (52.0)
Education, *n* (%)			
Junior high school	1 (8.3)	1 (7.7)	2 (8.0)
High school/vocational	6 (50.0)	1 (7.7)	7 (28.0)
Bachelor’s degree	4 (33.3)	6 (46.2)	10 (40.0)
Master’s degree	0 (0.0)	3 (23.1)	3 (12.0)
PhD degree	1 (8.3)	2 (15.4)	3 (12.0)
Occupation, *n* (%)			
Employee	7 (58.3)	5 (38.5)	12 (48.0)
Government agencies	4 (33.3)	7 (53.8)	11 (44.0)
Self-employed	0 (0.0)	1 (7.7)	1 (4.0)
Unemployed/Retired	1 (8.3)	0 (0.0)	1 (4.0)
Currently smoke, *n* (%)	2 (16.7)	2 (15.4)	4 (16.0)

**Table 2:** Blood biomarkers of participants in the watercress and placebo groups at baseline and EOT.

		Watercress Group (*n* = 12)	Placebo Group (*n* = 13)	*p*-Value ^b^
		Median (IQR)	Median (IQR)	
Fasting blood glucose, FBG (mg/dL)	Baseline	104.0 (93.8–107.8)	96.00 (92.5–104.5)	0.461
	EOT	102.0 (98.3–107.8)	96.00 (92.5–104.0)	0.155
	*p*-value ^a^	0.504	0.364	
Total cholesterol, TC (mg/dL)	Baseline	236.5 (199.8–262.3)	222.0 (202.5–246.0)	0.640
	EOT	221.0 (203.3–264.3)	194.0 (176.0–225.5)	0.123
	*p*-value ^a^	0.312	0.002 *	
Triglyceride, TG (mg/dL)	Baseline	142.0 (80.5–203.8)	105.0 (73.0–141.0)	0.220
	EOT	132.0 (86.3–155.0)	104.0 (69.0–134.5)	0.470
	*p*-value ^a^	0.196	0.853	
Low-density lipoprotein cholesterol, LDL-C (mg/dL)	Baseline	166.0 (128.5–191.0)	148.0 (134.5–201.0)	0.979
	EOT	175.5 (143.8–195.3)	145.0 (128.5–175.0)	0.172
	*p*-value ^a^	0.014 *	0.225	
High-density lipoprotein cholesterol, HDL-C (mg/dL)	Baseline	45.5 (40.3–51.0)	49.0 (43.0–56.5)	0.200
	EOT	47.5 (42.3–53.0)	50.0 (39.5–50.5)	0.989
	*p*-value ^a^	0.369	0.114	

Abbreviations: EOT, end of treatment; IQR, interquartile range. ^a^ The comparison between baseline data and EOT data in each group was analyzed by the Wilcoxon signed-rank test. ^b^ The comparison across two groups was analyzed by the Mann–Whitney U test. * Significantly different (*p*-value < 0.05).

## Data Availability

The data presented in this study are available on request.

## Abbreviations

The following abbreviations are used in this manuscript:

ABI	Ankle-brachial index
ABTS	2,2′-azino-bis-(3-ethylbenzothiazoline-6-sulfonic acid)
ACE	Angiotensin-converting enzyme
ARE	Antioxidant response element
ASVD	Atherosclerotic vascular disease
BMI	Body mass index
BP	Blood pressure
CAVI	Cardio-ankle vascular index
CI	Confidence interval
CVD	Cardiovascular disease
DBP	Diastolic blood pressure
EOT	End of treatment
FBG	Fasting blood glucose
GCL	Glutamate-cysteine ligase
GI	Gastrointestinal
GSH-Px	Glutathione peroxidase
HDL-C	High-density lipoprotein cholesterol
HEC	Human Experimentation Committee
HO-1	Heme oxygenase-1
HPLC	High-performance liquid chromatography
HUVEC	Human umbilical vein endothelial cell
IQR	Interquartile range
LDL-C	Low-density lipoprotein cholesterol
NO•	Nitric oxide
NO_2_^−^	Nitrite
NO_3_^−^	Nitrate
NQO1	NAD(P)H:quinone oxidoreductase
Nrf2	Nuclear factor erythroid 2-related factor 2
oxLDL	Oxidized low-density lipoprotein
P	Placebo
PEITC	Phenethyl isothiocyanate
PFV	Powerhouse fruit and vegetable
RAAS	Renin–angiotensin–aldosterone system
ROS	Reactive oxygen species
SBP	Systolic blood pressure
SD	Standard deviation
SOD	Superoxide dismutase
TC	Total cholesterol
TE	Trolox equivalent
TG	Triglyceride
W	Watercress
